# Perception of High School Students on risk for acquiring HIV and utilization of Voluntary Counseling and Testing (VCT) service for HIV in Debre-berhan Town, Ethiopia: a quantitative cross-sectional study

**DOI:** 10.1186/1756-0500-7-518

**Published:** 2014-08-12

**Authors:** Solomon Sisay, Woldaregay Erku, Girmay Medhin, Desalegn Woldeyohannes

**Affiliations:** Department of Biomedicine, School of Health Science, Dilla University, P.O. Box 419, Dilla, Ethiopia; Department of Tropical and Infectious Diseases, Aklilu Lemma Institute of Pathobiology, Addis Ababa University, P.O. Box 1176, Addis Ababa, Ethiopia; Department of Public Health, School of Medicine and Health Sciences, Addis Ababa Science and Technology University, P.O. Box 16417, Addis Ababa, Ethiopia

**Keywords:** Students, Risk perception, VCT use, HIV, Debre-berhan, Ethiopia

## Abstract

**Background:**

Human immunodeficiency virus (HIV) epidemic among youth is largely ignored and remains invisible to both young people themselves and to the society as a whole. Thus, the aim of the study was to assess the extent of perception risk of HIV and utilization of voluntary counseling and testing (VCT) service among high school students at Debre-berhan Town, Amhara Regional State, Ethiopia.

**Methods:**

A cross-sectional study was carried out from November 2010 up to January 2011 among secondary school students at Debre-berhan Town. Perception risk and VCT use were considered as dependant variables. A stratified random sampling technique was used to recruit study participants by taking schools as strata. Semi-structured self-administered questionnaire was used to collect the necessary data. Data was entered and analyzed using SPSS version 17.0. P-value < 0.05 was considered as statistically significant.

**Results:**

A total of 339 students were consented to participate in the study and the response rate was 96.3%. The student ages’ were ranged from 15 up to 24 years. Among the study participants, 30 (8.8%) had sexual contact and the mean age of first sexual encounter was 16.4 (SD =2.05) years. Of sexually active students, 12 (40%) had sex with different persons within the last 6 months, 13 (43.3%) had ever used condom and 15 (50%) had used VCT service. There was no statistically significant association between risk perception towards HIV infection and ever use of VCT service (AOR (95% CI) = 1.0(0.30, 4.02).

**Conclusions:**

Some students were engaged in risky sexual behavior even though they had heard about HIV/AIDS. The perception of risk for acquisition of HIV infection and utilization of VCT were low. Thus, education on topic of HIV/AIDS through integrating as part of school curriculum and encouraging the existing health institutions to provide youth-friendly sexual counseling services including VCT for HIV are strongly recommended.

## Background

Globally, an estimated 35 million people were living with human immunodeficiency virus (HIV) in 2012. At the same time, there were 2.3 million new HIV infections which showed a reduction by 33% as compared from 3.1 million new infections in 2001. Similarly, the number of HIV related deaths was also reduced up to 1.6 million in 2012 as compared from 2.3 million in 2005 [1].

In spite of improved access to antiretroviral treatment and care in many regions of the world, sub Saharan African countries took their greatest share for HIV epidemic [2]. Young people are particularly vulnerable to HIV pandemic and more than half of all new infections worldwide occur between the ages of 15 and 24 [3].

HIV/acquired immunodeficiency syndrome (AIDS) epidemic among youth is largely ignored and remains invisible to both young people themselves and to society as a whole so that the young are more likely to carry the virus for years without knowing that they are infected. Consequently, the epidemic spreads beyond high risk groups to the broader population due to which HIV/AIDS control programs become harder to succeed. Current data indicates that about 20% of young people in Africa, whose age range from 15 up to 19 years (mainly secondary school students), are infected with HIV virus [4].

A study which was done in Nigerian youth in 2007 revealed that about 43% of young people have had sex by the age of 15 and nearly 70% of sexual activity among them was unprotected [5]. In the same study, over one-third of boys and young men had slept with two or more partners in the previous three month. Additionally, the study revealed that about 90% of youth were unable to name all three principal way of avoiding HIV. Only 35% of them who knew that condom is protective used it one last time during sex as compared with 19% of those who did not know. Over one-third of them did not know where to buy condoms. Only 9% of them thought they were at high risk of contracting HIV; however, majority of the youth (86%) did not think they were at significant risk, ether because they explicitly thought they were safe or else they do not know about HIV/AIDS. Out of 70% of youth, who would like to have an HIV test, only 6% have had done the test.

These and other similar studies [3–5] indicated that still more work remain on awareness and behavioral changes among the most vulnerable section of the society, the youth. This was due to the fact that the number of youth who become infected with HIV was significantly increasing which accounts more than 50% of new HIV infection at global level as compared to other segments of risky population groups.

In absence of an effective vaccine and cure, voluntary counseling and testing service has been used as an entry point for behavioral change and access to antiretroviral treatment. Moreover, voluntary counseling and testing (VCT) is helping in the prevention of HIV transmission from mother-to-child with antiretroviral drugs through modification of infant feeding practice [2]. When HIV test was developed, it was intended to be accompanied by HIV counseling service. However, with growing awareness of HIV/AIDS and recent availability of antiretroviral therapy (ART), the scope of and reasons for voluntary counseling and HIV testing services have been broadened. VCT is a process by which an individual undergoes counseling to enable her/him to make an informed decision about being tested for HIV, assess their personal risk for HIV and develop a risk reduction strategy [6]. The service is essential components of HIV prevention and care programs. However, initially many young people were reluctant to be tested even if care and treatment were made available to them [6].

HIV voluntary counseling and testing service (VCT) is now widely accepted as the cornerstone of HIV prevention program in many countries because of its multiple benefits [7]. Unfortunately, many people including the young do not seek the services until they develop symptoms of AIDS [4]. Among the youth, barriers to use VCT service include lack of information, perception of low risk, lack of privacy and confidentiality, costs and laws that require parental consent [4].

In Ethiopia, HIV counseling and testing (HCT) begun few years before with service expanding throughout the country and it was reported that many young people with HIV in Ethiopia did not know that they were infected with the virus [8]. On the other hand, in 2011, Ethiopian Demographic Health Survey (EDHS) revealed that the overall prevalence of HIV in Ethiopia was estimated to be around 1.5% among the population whose age groups in between 15 up to 49 years [9]. Besides, women had a higher HIV prevalence (1.9%) as compared to men (1.0%). At the same time, the survey mentioned that 24% of women and 34% of men, whose age in between 15 up to 24 years, had comprehensive knowledge about HIV/AIDS. Their level of knowledge increased steadily with their educational status, but their main sources of information were believed to be from mass media, health professionals and schools. Similarly, 25% of young women and 28% of men, who had had sexual intercourse, had been tested for HIV. Another study conducted among high school students in Ethiopia showed that 62.2% of them supported the use of condom during sexual intercourse, but only 42.2% of students reported ever to have used condom during their first sexual encounter, while only 27.7% mentioned that they used condom every time [10].

The current study was different from the other studies conducted in Ethiopia, because it was mainly focused on assessing the students’ personal believe of HIV infection and their associated risk factors with comparisons of HIV testing. Hence, the objectives of the study were to identify students’ perception towards their susceptibility of HIV infection as well as their tendency on utilizing VCT and its barriers to the service.

## Methods

### Study design and area

A cross-sectional survey was conducted among high school students from November 2010 up to January 2011 at Debre-berhan Town, Amhara Regional State, Ethiopia. Ethiopia consisted of 9 regional states and 2 city administrations. Amhara Regional States had a total of 706 high schools [11]. Debre-berhan was one of the oldest towns within the region and located at 130 km towards the North East of Addis Ababa, the capital city of Ethiopia. Size of population in the town, for the year 2007 national census, was about 65,231. Out of which, 31,668 (48.5%) were males and 33,563 (51.5%) females [11]. There were one public high school and preparatory school in the town. High school contained from grade 9 up to grade 10, while preparatory school was from grade 11 up to grade 12.

### Study population

Debre-berhan high school students of both sexes from the two schools were used as source population.

### Exclusion criteria

Students who were not attending their class during the data collection period, evening students (those who were learning in the evening) and those who were not willing to take part in the study were not included.

### Sample size estimation

The required simple size was determined using  where p is prevalence of the intention to get HIV test, Δ is margin of error between the sample estimate and true population value and z is critical value corresponding to a given level of confidence. Assuming 95% confidence level and 4% margin of error [12] and estimated demand of 85% for VCT based on previous Ethiopian study [12], the required minimum sample size was 306. With additional assumption of 15% non-response rate, the overall sample size was 352.

### Sampling techniques

A stratified random sampling technique was used to recruit the required number of students from the two secondary schools in Debre-berhan Town where the strata were grade 9, 10, 11 and 12. Students that correspond to a given grade were selected through simple random sampling technique using the school roster as a sampling frame.

### Sampling procedure

High schools consisted of grades 9, 10, 11 and 12. Each of which having several study populations were categorized from source population based on the level of the grade. The numbers of study subjects included in each grade were proportional to their size. Then, sections to be included in each grade were selected based on simple random sampling. The students from the selected sections were assembled in a room and requested to fill out questionnaire in the presence of data collectors. Data collection process was monitored by the two trained data collectors.

### Instrument of data collection

Data was collected using semi-structured self-administered questionnaire. The questionnaire was prepared first in English and it was then translated in to Amharic which was the official language of the study area. Amharic version of the questionnaire was pre-tested for clarity, acceptability and flow among non-study subjects before actual data collection and necessary adjustments were made. The questionnaire was adapted from HIV/AIDS behavioral surveillance survey (BSS) of Ethiopia [8]. A total of 44 questions were involved in the questionnaire. Most of questions were linked to demographic characteristics, perception risk including knowledge, attitude, practice of HIV/AIDS and VCT of the students.

### Data analysis

The questionnaire which was completed by the study participating students was coded and computerized using SPSS version 17.0 packages. Students were classified as being knowledgeable about HIV transmission if they were able to provide correct response to all four questions describing means of HIV transmission (i.e. mother-to-child, blood transfusion, sexual intercourse and injury with sharp contaminated materials). Data was summarized using frequencies and percentages. Logistic regression was used to adjust for possible confounding factors. Results were reported statistically significant if P-value was less than 0.05.

### Dependent variables

Perception risk and VCT use were considered as dependent variables.

### Independent variables

Age, sex, marital status, level of education, use of condom, ever practice sex, VCT use were considered as independent variables.

## Operational definitions

### Youth

Those who are in the age group from 15 up to 24 years.

### Perception

A reception and interpretation of sensory input related to HIV preventive method.

### Risk

A situation in which an action will result in an outcome that is not known with certainty, but the set of possible outcomes and their associated probabilities are known or can be estimated.

### Behavior

Various voluntary movements undertaken by the body in response to motives and decision related to HIV preventive methods.

### Perception risk

Students’ attitude towards perceiving themselves as susceptible to HIV infection.

### High risk perception

Students were considered to have high perception risk if they had been exposed to at least one of the conditions like having sex without condom, having sex with prostitution, having sex with HIV infected person and having injury with HIV infected sharp materials.

### Low risk perception

Students were considered to have low perception risk if they had no sexual contact, if they had used condom during sexual intercourse and being faithful to their sexual partners.

## Ethical considerations

Ethical clearance was obtained before data collection from Institutional Review Board of Aklilu Lemma Institute of Pathobiology, AAU. Then, formal letter was written to Debre-berhan Woreda Educational Bureau and the two targeted high schools. Consent was requested from the students prior to the study there by informing them to participate or refuse in the study. No personal identification was recorded on the questionnaire for ethical reason.

## Results

### Socio-demographic characteristics

A total of 339 students participated in the study and the response rate was 96.3%. Out of the total respondents, 161 (47.5%) were males and the rest were females. The mean age of the study participants was 17.1 (SD = 2.1) years. Three hundred and thirty-four (98.5%) of the respondents were never married. The majority of the respondents 330 (97.3%) were Amhara by ethnicity and 321 (94.7%) were Orthodox Christians followed by Protestants 9 (2.7%). Among the total respondents, 240 (70.8%) were from grade 9 up to grade 10 and the rest were from grade 11 up to grade 12 students (Table 1).

**Table 1:** **Socio-demographic characteristics of high school students in Debre-berhan Town, January 2010**

Variables	Number	Percent (%)
Age		
15-19	308	90.9
20-24	31	9.1
Sex		
Male	161	47.5
Female	178	52.5
Residence		
Urban	289	85.3
Rural	50	14.7
Marital status		
Single	334	98.5
Married	5	1.5
Ethnicity		
Amhara	330	97.3
Oromo	5	1.5
Tigre	1	0.3
Other	3	0.9
Educational status		
9-10	240	70.8
11-12	99	29.2

### Knowledge related to HIV/AIDS

**Figure 1:**
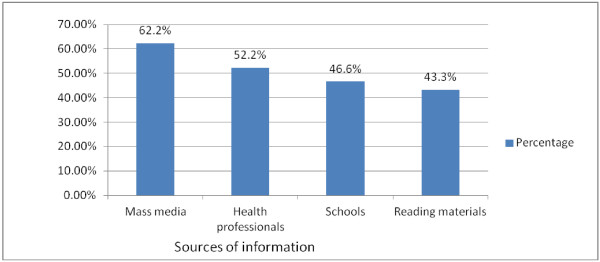
**Sources of information for HIV among high school students in Debre-berhan Town, January 2010.**

All respondents heard about HIV/AIDS, and the most frequently mentioned sources of information for HIV/AIDS were mass media 211 (62.2%) followed by health professionals 177 (52.2%) (Figure 1). Two hundred and seventy-four (80.8%) of students responded that HIV can be transmitted by sexual means whereas 217 (64%) of the respondents reported that it is possible for the virus to be transmitted from mother-to-child. Additionally, 236 (69.6%) and 200 (59%) of students said that the HIV can be transmitted through sharp injury with HIV infected blood materials and blood transfusion, respectively.

Condom use 295 (87%), abstinence 233 (68.7%), and being faithful to one partner 128 (37.8%) were mentioned as means of best preventing methods for HIV/AIDS. In addition, 299 (88.2%) of students mentioned that virginity should be encouraged as it is one of prevention method for HIV (Table 2). Knowledge on HIV transmission was inversely and significantly associated with educational status (P < 0.05). Students with high school level were less knowledgeable about HIV transmission compared to students who were in preparatory level (AOR (95% CI) =0.3 (0.2, 0.5) (Table 3).

**Table 2:** **Knowledge of students on modes of HIV transmission and best prevention method in Debre-berhan Town, January 2010**

Variable	M (%)	F (%)	Total (%)
Variable			
Yes	142 (41.9)	132 (38.9)	274 (80.8)
No	19 (5.6)	46 (13.6)	65 (19.2)
Mother-to-fetus			
Yes	125 (36.9)	92 (27.1)	217 (64)
No	36 (10.6)	86 (25.4)	122 (36)
Insect bite transmit HIV			
Yes	50 (14.7)	87 (25.7)	137 (40.4)
No	111 (32.7)	91 (26.8)	202 (59.6)
Kissing transmit HIV			
Yes	34 (10)	68 (20.1)	102 (30.1)
No	127 (37.5)	110 (32.4)	237 (69.9)
Abstinence protects from HIV			
Yes	121 (35.7)	112 (33)	233 (68.7)
No	40 (11.8)	66 (19.5)	106 (31.3)
Faithfulness protects from HIV			
Yes	51 (15.1)	77 (22.7)	128 (37.8)
No	110 (32.4)	101 (29.8)	211 (62.2)
Condom use protects from HIV			
Yes	147 (43.4)	148 (43.7)	295 (87)
No	14 (4.1)	30 (8.9)	44 (13)

*NB: Percents will not add up to 100, as multiple responses are possible.*

**Table 3:** **Correlates of knowledge on HIV transmissions with selected variables among high school students in Debre-berhan Town, January 2010**

Variables	Knowledge on HIV		OR (95% CI)	
	Yes	No	Crude	Adjusted*
Sex				
Male	97	64	0.5 (0.2, 0.7)	0.6 (0.4, 1.0)
Female	73	105	1.0	1.0
Age				
15-19	149	159	0.3 (0.5, 0.7)	0.6 (0.3, 1.6)
20-24	21	10	1.0	1.0
Educational status				
9-10	92	148	0.2 (0.1, 0.4)	0.3 (0.2, 0.5) **
11-12	78	21	1.0	1.0
Marital status				
Single	168	166	1.5 (0.3, 9.2)	1.3 (0.2, 11.0)
Married	2	3	1.0	1.0
Ever practiced sex				
Yes	10	20	1.2 (0.6, 2.4)	1.0 (0.4, 2.5)
No	160	149	1.0	1.0
Risk perception				
Low	167	156	2.0 (0.6, 5.7)	2.0 (0.5, 7.2)
High	3	13	1.0	1.0
Willingness to VCT				
Yes	149	141	0.9 (0.5, 1.7)	0.8 (0.4, 1.6)
No	21	28	1.0	1.0

NB**=significant (P<0.05).

Adjusted*: for sex, age, educational status, marital status, ever practiced sex, risk perception, willingness to VCT.

Discrepancies had been observed among the respondents’ knowledge and their practices. Only 7 (23.3%) of those respondents who ever had sex believed appropriate use of condom can be protective against HIV and 13 (43.3%) of them did use condom during last sexual intercourse. Eighteen (60%) of those who ever had sex knew that abstaining can protect people from HIV infection. Similarly, 15 (50%) of those who had sex knew that faithfulness can protect people from HIV infection and 12 (40%) of them were not faithful during sexual activity.

### Risk perception on HIV/AIDS

Participants’ perception on their susceptible to HIV infection was asked and the result indicated that 323 (95.3%) and 16 (4.7%) believed to have low and high chances of acquiring the virus, respectively. The proportions of students who perceived themselves at risk of contracting HIV were not similar for both sexes. Among those who perceived themselves at risk, 12 (3.5%) reported that they had sex without condom, 2 (0.6%) had injury with contaminated materials, 1 (0.3%) had sex with prostitute, 1 (0.3%) had sex with HIV positive person. The most frequently cited reasons by those who did not perceive themselves at risk were absence of sexual contact 309 (91.2%) followed by using condom 13 (3.8%) and faithful to one partner 12 (3.5%) (Table 4).

**Table 4:** **Proportion of students by HIV risk perception and reasons for risk perception in Debre-berhan Town, January 2010**

Variable	M (%)	F (%)	Total (%)
Risk perception			
Low	154 (45.4)	169 (49.9)	323 (95.3)
High	7 (2)	9 (2.7)	16 (4.7)
Reasons for risk			
Had sex without condom	4 (1.2)	8 (2.4)	12 (3.6)
Injury with contaminated material	2 (0.6)	------	2 (0.6)
Had sex with prostitution	1 (0.3)	------	1 (0.3)
Had sex with HIV positive person	-----	1 (0.3)	1 (0.3)
Reasons for not at risk			
Never had sex	144 (42.5)	165 (48.7)	309 (91.2)
Use condom	11 (3.2)	2 (0.6)	13 (3.8)
Faithful to partner	6 (1.8)	6 (1.8)	12 (3.6)

*NB: Percents will not add up to 100, as multiple responses are possible.*

Risk perception of HIV was significantly associated with those students who had ever practiced sex (AOR (95% CI) =0.02(0.01, 0.1). Those students who ever had practiced sex perceived themselves less frequently at low risk for acquiring HIV as compared those who didn’t practice sex while variables like sex, age, educational status, marital status, knowledge on HIV transmission, and ever used VCT were not significantly associated with risk perception to HIV (P > 0.05) (Table 5).

**Table 5:** **Correlates of risk perception with selected variables among high school students in Debre-berhan Town, January 2010**

Variables	Risk perception		OR (95% CI)	
	Low	High	Crude	Adjusted*
Sex				
Male	154	7	0.6 (0.2. 1.9)	0.6 (0.1, 2.3)
Female	169	9	1.0	1.0
Age				
15-19	294	14	0.7 (0.1, 3.1)	1.0 (0.2, 6.2)
20-24	29	2	1.0	1.0
Educational status				
9-10	226	14	1.6 (0.4, 5.7)	1.7 (0.3, 9.2)
11-12	97	2	1.0	1.0
Marital status				
Single	320	14	0.2 (0.02, 1.6)	1.3 (0.1, 17.8)
Married	3	2	1.0	1.0
Knowledge on HIV transmission				
Yes	167	3	2.0 (0.6, 5.7)	2.0 (0.5, 7.9)
No	156	13	1.0	1.0
Ever practiced sex				
Yes	16	17	0.02 (0.01, 0.07)	0.02 (0.01, 0.1)**
No	307	2	1.0	1.0
Ever used VCT				
Yes	124	6	0.9 (0.3, 2.6)	1.1 (0.3, 4.1)
No	199	10	1.0	1.0

NB**=significant (P<0.05).

Adjusted*: for sex, age, educational status, marital status, knowledge of HIV transmission, ever practiced sex, ever used VCT.

### Sexual behavior of students

Thirty (8.8%) of the respondents had sexual experience. Out of which, 16 (4.7%) were females and the rest 14 (4.1%) were males. Age at first sexual contact ranged from 15 up to 19 years. The mean age of first sexual contact was being 16.4 (SD = 2.0) in years. With regard to protecting oneself from HIV infection, 309 (91.2%) of the students claimed that they were abstaining from sex, 13 (3.8%) of them were using condoms for HIV protection (Table 6).

**Table 6:** **Sexual history profile of high school students in Debre-berhan Town, January 2010**

Variables	M (%)	F (%)	Total (%)
Ever practiced sex			
Yes	17 (5)	13 (3.8)	30 (8.8)
No	144 (42.5)	165 (48.7)	309 (91.2)
Had multiple sexual contact within 6 months			
Yes	10 (2.9)	2 (0.6)	12 (3.5)
No	151 (44.5)	176 (52.1)	327 (95.6)
Methods of HIV protection			
Abstain	144 (42.5)	165 (48.7)	309 (91.2)
Use condom	11 (3.2)	2 (0.6)	13 (3.8)
Faithful to partner	6 (1.8)	6 (1.8)	12 (3.6)

*NB: Percents will not add up to 100, as multiple responses are possible.*

Two hundred and forty-three (71.7%) of students revealed that they had not regular friend while 96 (28.3%) reported that they had. Out of those who had regular friend, 15 (4.4%) of respondents claimed that they had sex with their friend and the rest 81 (23.9%) of respondents haven’t had sex. The main reasons cited by the students for not having sex with regular friend were decision not to do it before marriage 53 (15.6%), decision not to do it before HIV test 18 (5.3%), to avoid pregnancy 7 (2.1%) and to prevent oneself from sexually transmitted infection (STI) 3 (0.9%).

Out of those who responded to have had previous history of sexual contact, 5 (16.7%) of males and 7 (23.3%) of females had multiple sexual contact during the last six month. Out of those who had multiple sexual contact, 3 (25%) of them said that they had sexual contact with more than four partners during the same period. The main reasons cited by students for having multiple sexual partners include due to sexual desire, 9 (75%); due to cultural reason, 1 (8.3%); and due to seeking to have more children1 (8.3%); and economic reasons 1 (8.3%).

Variables such as sex, age, educational level, knowledge on HIV transmission, and willingness to use VCT did not show significant association (P > 0.05) with ever practiced sex, where as risk perception showed significant association (P < 0.05) with ever practiced sex which was a negative association. Those students who perceived themselves at low risk were less frequently engaged in sexual practice than those students who perceived themselves at high risk (AOR (95% CI) =0.02(0.001, 0.1) (Table 7).

**Table 7:** **Correlates of sexual behavior with selected variables among high school students in Debre-berhan Town, January 2010**

Variables	Ever practiced sex		OR (95% CI)	
	Yes	No	Crude	Adjusted*
Sex				
Male	17	144	0.8 (0.4, 1.6)	0.9 (0.4, 2.1)
Female	13	165	1.0	1.0
Age				
15-19	23	285	0.4 (0.2, 1.1)	0.4 (0.1, 1.3)
20-24	7	24	1.0	1.0
Educational status				
9-10	22	218	1.1 (0.5, 2.4)	1.3 (0.5, 3.9)
11-12	8	91	1.0	1.0
Knowledge on HIV transmission				
Yes	10	160	1.2 (0.6, 2.4)	1.0 (0.4, 2.5)
No	20	149	1.0	1.0
Risk perception				
Low	16	307	0.02 (0.01, 0.1)	0 .02 (0.01, 0.1)**
High	14	2	1.0	1.0
Willingness to use VCT				
Yes	21	269	2.0 (1.0, 4.5)	1.2 (0.4, 3.6)
No	9	40	1.0	1.0

NB**=significant (P<0.05).

Adjusted*: for sex, age, educational status, marital status, knowledge on HIV transmission, risk perception, *willingness to use VCT.*

### Knowledge, attitude and practice towards VCT service

**Figure 2:**
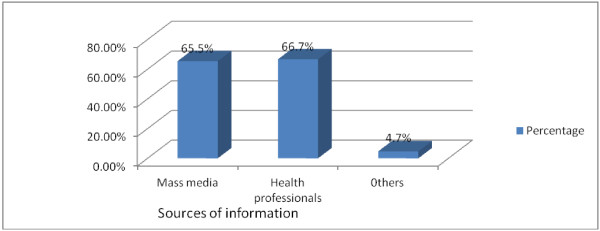
**Sources of information for VCT among high school students in Debre-berhan Town, January 2010.**

All of the respondents reported that they had heard about VCT from different sources. Health professionals were the main source of information to access for the majority 226 (66.7%) of the respondents (Figure 2). When asked whether VCT was necessary or not, 327 (96.5%) of the respondents thought it was necessary. Concerning the preference way of receiving HIV test, majority 286 (84.4%) preferred face-to-face followed by secretive letter 44 (13%).

**Figure 3:**
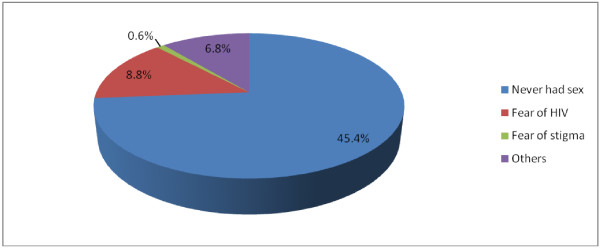
**Reasons for not using VCT by percentage among high school students in Debre-berhan Town, January 2010.**

Regarding their history of using VCT, 130 (38.3%) used VCT service, out of whom, male and female accounted for 62 (18.3%) and 68 (20%), respectively (Table 6). Majority of the respondents who had used VCT service responded that their reasons to get tested were for their desire to know their sero-status 124 (36.6%) while 6 (1.8%) did it for marriage purpose. The rest 209(61.7%) of the respondents who do not use VCT claimed that their reasons for not taking part in VCT were never had sex 154 (45.4%), due to fear of having the virus 30 (8.8%) and 2 (0.6%) were due to fear of stigma and discrimination by the society (Figure 3).

Students’ willingness to undertake VCT was assessed. Two hundred-ninety 290 (85.5%) of the respondents responded that they were willing while the rest 49 (14.5%) were not (Table 8). Discrepancies had been observed among the respondents’ knowledge and their practices. Forty-two (12.4%) of those who felt VCT is necessary were not willing to use VCT service. Similarly, of those respondents who believed VCT is necessary, 200 (59%) did not actually use VCT service.

**Table 8:** **Knowledge, attitude and practice of Debre-berhan high school students towards VCT, January 2010**

Variables	M (%)	F (%)	Total (%)
Heard of VCT			
Yes	161 (47.5)	178 (52.5)	339 (100)
No	-------	-------	-------
Feel that VCT necessary			
Yes	155 (45.7)	172 (50.7)	327 (96.4)
No	6 (1.8)	6 (1.8)	12 (3.6)
Willingness to use VCT service			
Yes	139 (41)	151 (44.5)	290 (85.5)
No	22 (6.5)	27 (7.8)	49 (14.5)
Used VCT service			
Yes	61 (18)	69 (20.4)	130 (38.4)
No	100 (29.4)	109 (32.2)	209 (61.6)
Availability of VCT service in the vicinity			
Yes	131 (38.6)	152 (44.8)	283 (83.5)
No	30 (8.8)	26 (7.7)	56 (16.5)

*NB: Percents will not add up to 100, as eight students failed to answer above variables.*

With regard to timing for testing, majority of the students 292 (86.1%) said that one should undergo VCT at any time, 22 (6.5%) agreed just before marriage, 14 (4.1%) thought when an individual having many sex partner, and 7 (2.1%) thought when an individual falls ill. Students’ belief towards provision of safety for other by having HIV test was also assessed. Two hundred seventy-three (80.5%) of respondents agreed that HIV test provide safety for other while 66 (19.5%) of them disagreed. Moreover, 99 (29.2%) of respondents who had used VCT believed that HIV test helps in alleviating anxiety and the rest 31 (9.1%) disagreed.

Availability of VCT service in the vicinity of students’ was also assessed. Two hundred eighty- three (83.5%) of respondents claimed that they could access VCT service in their nearby area where as 56 (16.5%) of them responded that they could not access the service (Table 8). Condom use was strongly correlated and significantly associated with ever used VCT (AOR (95% CI) =4.5 (1.1, 23.3) which was a positive association. Those students who used condom are more likely to be tested for HIV as compared to those who didn’t use (P < 0.05) (Table 9).

**Table 9:** **Association of socio-demographic variable and practice with ever VCT service use among high school students in Debre-berhan Town, January 2010**

Variables	Ever used VCT		OR (95% CI)	
	Yes	No	Crude	Adjusted*
Age				
15-19	114	194	0.6 (0.3, 1.3)	0.6 (0.3, 1.3)
20-24	16	15	1.0	1.0
Sex				
Male	61	100	1.1 (0.7, 1.7)	1.1 (0.7, 1.8)
Female	89	109	1.0	1.0
Marital status				
Single	124	208	0.2 (0.02, 1.5)	0.1 (0.01, 1.5)
Married	4	1	1.0	1.0
Educational status				
9 - 10	93	147	1.1 (0.7, 1.8)	1.2 (0.7, 2.1)
11-12	37	62	1.0	1.0
Ever had sex				
Yes	15	15	0.7 (0.4, 1.5)	2.2 (0.5, 9.0)
No	115	194	1.0	1.0
Condom use				
Yes	6	7	2.3 (0.8, 6.1)	4.5 (1.1, 23.3)**
No	134	192	1.0	1.0
Risk perception				
Low	124	199	0.9 (0.3, 2.6)	1.0 (0.3, 4.02)
High	6	10	1.0	1.0

NB** *= significant (P<0.05).*

Adjusted*: for age, sex, marital status, education, ever practiced sex, condom use and risk perception.

## Discussion

All students had heard about HIV/AIDS, its transmission and prevention which was similar to the findings of a study carried out in Nigeria [5, 13]. However, this level of awareness did not necessarily reflect the understanding of how HIV can be transmitted or prevented. Even though students were well informed about HIV, there was knowledge gap on transmission and protective practice. In this study, only 170 (50.1%) could mention all possible ways of HIV transmission and only 30 (8.8%) could identify all possible methods of protecting themselves against HIV infection. This low level of knowledge could predispose these young people to HIV infection.

In this study, 30 (8.8%) of the respondents (14 (4.1%) males and 16 (4.7%) females) had sexual experience which was a bit lower as compared to other study in Ethiopia where 33.3% of students reported that they had sexual intercourse for the first time with the mean age of 15.3 years [14]. Another study conducted on determining perception risk for HIV among adolescent in Uganda showed that 52.2% of boys and 48.8% of girls had sexual experience with the mean age of sexual commencement being 17 years [15]. These might be explained due to the fact that students might not tell the truth because of the sensitivity of the issue in the context of culture of society or might be associated with students’ determination of abstaining from sex as a means of prevention for HIV.

Among students who had sex, 13 (43.3%) were using condoms for HIV protection. This was lower condom use rate as compared to other findings elsewhere, which puts the students at high risk of acquiring sexually transmitted including HIV/AIDS. A study conducted on high risk sexual behavior among youth in Tanzania revealed that 49% of youth reported to have used condom [16]. Another study done among youth in Addis Ababa indicated that condom use rate was found to be up to 48% [6]. But, the finding was comparable to behavioral surveillance survey (BSS) report in Ethiopia [8] which showed that among in-school students who reported to have had sex, 43.1% of them had used condom.

These findings showed that there was still a gap of knowledge in the preventive methods of HIV. This low level of condom usage might indicate that the students might not easily get access to condom due to unavailability of service provider in nearby area or might have embarrassed to go and ask for condom or might be related to the expense of condom.

Majority of students 323 (95.3%) perceived themselves of being at low risk of getting HIV. A similar study in Northwest Ethiopia revealed that only 18.6% of students felt that they could have a high chance for acquiring HIV [17]. Another study which was done in Cape Town revealed that the majority of the youth perceived themselves as being at a little or no risk of HIV infection [18]. The reasons for such low risk perception among students could be that students may underestimate risks in general because of feeling of invulnerability; and that HIV is highly stigmatized by the community so that accepting the risk leads to the possibility of being isolated there by preventing to believe their own personal risk.

All of the study participants (100%) heard the existence of VCT service from different sources. However, only 130 (38.3%) of the study participants (62 (18.2%) males and 68 (20.1%) females) ever used VCT. Although there was apparent discrepancy between awareness and use of VCT service among the study participants, the findings were higher than 9.3% which was reported by BSS in 2005 [8], for VCT use among in-school youth. This showed an increment in the acceptance of VCT as preventive measure. The reason for this improvement could be either positive behavioral changes or expansion of VCT service in the country in the last few years.

The existence of 49 (14.5%) of unwilling students to use VCT service showed the importance of continuing advocacy activities that promote its use. Abstaining from sex, fear of being positive for the test and fear of stigma and discrimination by community were important factors mentioned by the students to refrain them from using VCT service. A study in Botswana also identified fear of the virus, lack of service and stigma associated with HIV/AIDS as key factors in reducing the uptake of VCT services among young students [4].

The study revealed that the perception of being at risk and utilization of VCT were inversely associated. Significant number of students who perceived themselves at low risk for HIV infection had utilized VCT service compared to those who considered themselves to be at higher risk. Similarly, a significant number of those students who did not have sexual contact used HIV test better than those who had sexual experience. This finding was similar to the one obtained on risk perception of HIV infection and VCT utilization among Nigerian youth, which showed that low level of using VCT service was accompanied by high perception risk of infection among adolescents [5]. The result was also true in BSS report [8], which revealed that those students who perceived themselves to be at no or low risk of HIV infection were also more likely to be tested than those who perceive at high risk. This might be due to the fact that they were afraid that their test result could be HIV positive for they could have been engaged in unprotected sexual intercourse.

## Conclusions

All students had heard about HIV/AIDS, but misconceptions existed regarding HIV transmission and prevention. Students were engaged in unprotected sexual intercourse which was indicated by low condom use. The perception of majority of the students towards acquisition of HIV was low. Some of students thought that VCT was not necessary even though their knowledge about the benefit of VCT was high. In general, majority of students were willing to use VCT service except for some discouraging factors they mentioned such as fear of stigma, fear of being positive for the test and a misconception that use of VCT is unnecessary if a person is abstaining from sex. It would be advisable to include topics on HIV/AIDS in the school’s curriculum to update the knowledge of students as well as teachers. It is also important to integrate VCT service to anti-AIDS club in schools which assists in making the service to be expanded and easily accessible for students. Information, education and communication program to advocate the benefit of VCT and reduce fear of stigma and discrimination should be strengthened. Improving the existing health institution to provide youth-friendly sexual counseling services including VCT for HIV is also strongly recommended.

## Limitations of the study

In the study, the authors used quantitative data for analysis, but it lacked qualitative data which were helpful in substantiating quantitative findings. At the same time, the questionnaire contained culturally sensitive inquiry for the respondents in the study area, for example, about their sexual behavior which might influence the students to provide biased information. Non-respondent rates were not totally avoidable from the study. Moreover, the number of schools which were involved in the study was minimal. Therefore, the findings might be affected in terms of generalizability.

## Abbreviations

AAU: Addis Ababa University
AIDS: Acquired Immuno Deficiency Syndrome
AOR: Adjusted Odds Ratio
ART: Anti Retroviral Treatment
BSS: Behavioral Surveillance Survey
CI: Confidence Interval
EDHS: Ethiopian Demographic Health Survey
HCT: HIV Counseling and Testing
HIV: Human Immunodeficiency Virus
SD: Standard Deviation
SPSS: Statistical Package for Social Science
STI: Sexually Transmitted Infection
VCT: Voluntary Counseling and Testing for HIV.
